# Positive effect of deep diaphragmatic breathing training on gastroesophageal reflux-induced chronic cough: a clinical randomized controlled study

**DOI:** 10.1186/s12931-024-02783-5

**Published:** 2024-04-18

**Authors:** Shanshan Niu, Tongyangzi Zhang, Wanzhen Li, Siwan Wen, Lei Dong, Shengyuan Wang, Wenbo Shi, Cuiqin Shi, Yuqin Shen, Qianchun Huang, Yaling Tan, Xianghuai Xu, Li Yu

**Affiliations:** 1 Department of Pulmonary and Critical Care Medicine, Tongji Hospital, School of Medicine, Tongji University, No. 389 Xincun Road, Shanghai, 200065 China; 2 Department of Oncology, Shanghai Yangzhi Rehabilitation Hospital (Shanghai Sunshine Rehabilitation Center), School of Medicine, Tongji University, Shanghai, China; 3 Department of Allergy, Tongji Hospital, School of Medicine, Tongji University, No. 389 Xincun Road, Shanghai, 200065 China; 4 Department of Cardiac Rehabilitation, Tongji Hospital, School of Medicine, Tongji University, No. 389 Xincun Road, Shanghai, 200065 China; 5 Department of Neurology, Tongji Hospital, School of Medicine, Tongji University, No. 389 Xincun Road, Shanghai, 200065 China

**Keywords:** Deep diaphragmatic breathing training, Chronic cough, Gastroesophageal reflux, Diaphragm, Non-pharmacological treatment

## Abstract

**Background and Objective:**

To explore the efficacy of deep diaphragmatic breathing training (DEP) in patients with gastroesophageal reflux-induced chronic cough (GERC).

**Methods:**

A randomized controlled study was conducted involving 60 GERC patients who were divided into the intervention group and the control group (each with 30 patients). Both groups received routine medication treatment for GERC, while the intervention group received DEP training additionally. Both groups were evaluated by cough symptom scores, Hull airway reflux questionnaire (HARQ), gastroesophageal reflux diagnostic questionnaire (GerdQ), generalized anxiety disorder scale-7 (GAD-7), patient health questionnaire-9 (PHQ-9), Pittsburgh sleep quality index (PSQI), the Leicester cough questionnaire (LCQ), as well as capsaicin cough sensitivity testing, B-ultrasound and surface electromyography (sEMG) of the diaphragmatic muscles before and after treatment. The cough resolution rate and changes of the above indictors was compared between the two groups after eight weeks of treatment.

**Results:**

After eight weeks of treatment, cough symptoms improved in both groups, but the cough resolution rate in the intervention group of 94% was significantly higher than that in the control group of 77% (χ^2^ = 6.402, *P* = 0.041). The intervention group showed significant improvements to the control group in GerdQ (6.13(0.35) VS 6.57(0.77)), GAD-7 (0(0;1) VS 1(0;3)), PSQI (2(1;3) VS 4(3;6)), LCQ (17.19(1.56) VS 15.88(1.92)) and PHQ-9 (0(0;0) VS 0(0;3)) after treatment. Compared to control group, sEMG activity of the diaphragmatic muscle was significantly increased in the intervention group after treatment, measured during DEP (79.00(2.49) VS 74.65 (1.93)) and quiet breathing (72.73 (1.96) VS 67.15 (2.48)).

**Conclusion:**

DEP training can improve cough symptoms as an adjunctive treatment in GERC patients.

**Trial registration:**

The protocol was registered in February 2, 2022 via the Chinese Clinical Trials Register (http://www.chictr.org.cn/) [ChiCTR2200056246].

**Supplementary Information:**

The online version contains supplementary material available at 10.1186/s12931-024-02783-5.

## Introduction

Gastroesophageal reflux-induced chronic cough (GERC) is a common subtype of gastroesophageal reflux disease (GERD) characterized by chronic cough as the main symptom [1–3]. The incidence of GERC varies by region and accounts for 5 to 40% of the causes of chronic cough [3, 4]. With the deepening understanding, advances in examination methods and changes in lifestyles and dietary structure of GERC patients, the rate of GERC in China is increasing [5, 6]. Current guidelines in China recommend a standard anti-reflux treatment course of at least eight weeks, but 36% of patients still require the use of neuro regulators to improve treatment, which often results in side effects such as drowsiness and dizziness [7]. The treatment of GERC, therefore, remains challenging with significant impacts on patient’s quality of life and economic prospects [8, 9].

The main pathogenesis of GERD is the weakening of the anti-reflux barrier [10]. The high-pressure zone at the gastroesophageal junction, formed by the lower esophageal sphincter (LES), diaphragm and related structures, is a critical part of the anti-reflux barrier [11]. Once the function of the diaphragm and LES is impaired, the anti-reflux barrier weakens, leading to the occurrence of GERD.

Deep diaphragmatic breathing (DEP) training transforms chest breathing or mixed chest and abdominal breathing into DEP, using the contraction and relaxation of the diaphragm muscle to achieve deep and slow rhythmic breathing. Several recent studies have shown that DEP can improve symptoms in patients with chronic obstructive pulmonary disease by enhancing the function of the diaphragm [12, 13]. Eherer et al. found that DEP can improve the quality of life of GERD patients, reduce esophageal acid exposure time and hypothesized that DEP could enhance diaphragmatic muscle tension to strengthen the anti-reflux barrier and improve symptoms of gastroesophageal reflux [14]. The use of DEP can also enhance the pinchcock effect of the diaphragm on the LES, strengthening the anti-reflux barrier [11]. Since GERC is a subtype of GERD and the cough symptoms in GERC patients are also partially due to impaired anti-reflux barrier function, it is hypothesized that DEP may have value as a new safe and non-invasive auxiliary treatment option in GERC treatment.

This prospective randomized controlled study aimed to explore the effects of combining DEP with anti-reflux drug therapy compared to drug therapy alone on cough symptoms, reflux symptoms, quality of life as well as sleep and psychological conditions in GERC patients.

## Methods

### Subjects

This was a single-center, randomized, controlled prospective study that recruited suspected GERC patients who visited our department from August 2021 to December 2022. Complete medical history, physical examination, capsaicin cough sensitivity test, chest CT or X-ray examination, pulmonary function test, histamine bronchial provocation test, induced sputum cytology examination and multichannel intraluminal esophageal impedance and pH monitoring (MII-pH) data were collected. The research plan was approved by the Ethics Committee (2021-064) and registered in the Chinese Clinical Trial Registry. (ChiCTR2200056246). All study subjects were informed and signed informed consent forms.

The inclusion criteria included: ①suspected GERC, aged between 18 and 80 years, and had a cough course exceeding eight weeks; ②these patients had no obvious abnormalities on chest X-ray or chest CT images, pulmonary function with forced expiratory volume in one second/forced vital capacity (FEV1/FVC) exceeding 70%, percentage of predicted FEV1 value exceeding 80% of the expected value and ③were able to complete DEP training. ④ MII-pH where acid exposure time (AET) exceeded 6% and/or symptom association probability (SAP) exceeding 95% and/or symptom index (SI) exceeding 50%. The exclusion criteria included: ①pregnant or lactating women, smoking or smoking cessation of fewer than two years; ②abnormal moist rales on lung auscultation; ③symptoms such as fever, hemoptysis and dyspnea; ④who were unable to read and understand the questionnaire, refusal to sign the informed consent form. And, the patient who had incomplete data or were violated of the treatment plan and loss of follow-up would be excluded from analysis.

The GERC diagnosis criteria [2, 3, 9, 15, 16] included a cough duration exceeding eight weeks, with or without typical reflux symptoms such as acid regurgitation and heartburn, MII-pH where acid exposure time (AET) exceeded 6% and/or symptom association probability (SAP) exceeding 95% and/or symptom index (SI) exceeding 50% and cough responsive to a stepwise anti-reflux therapy (cough symptom score decreased by > 50%).

**Fig. 1:**
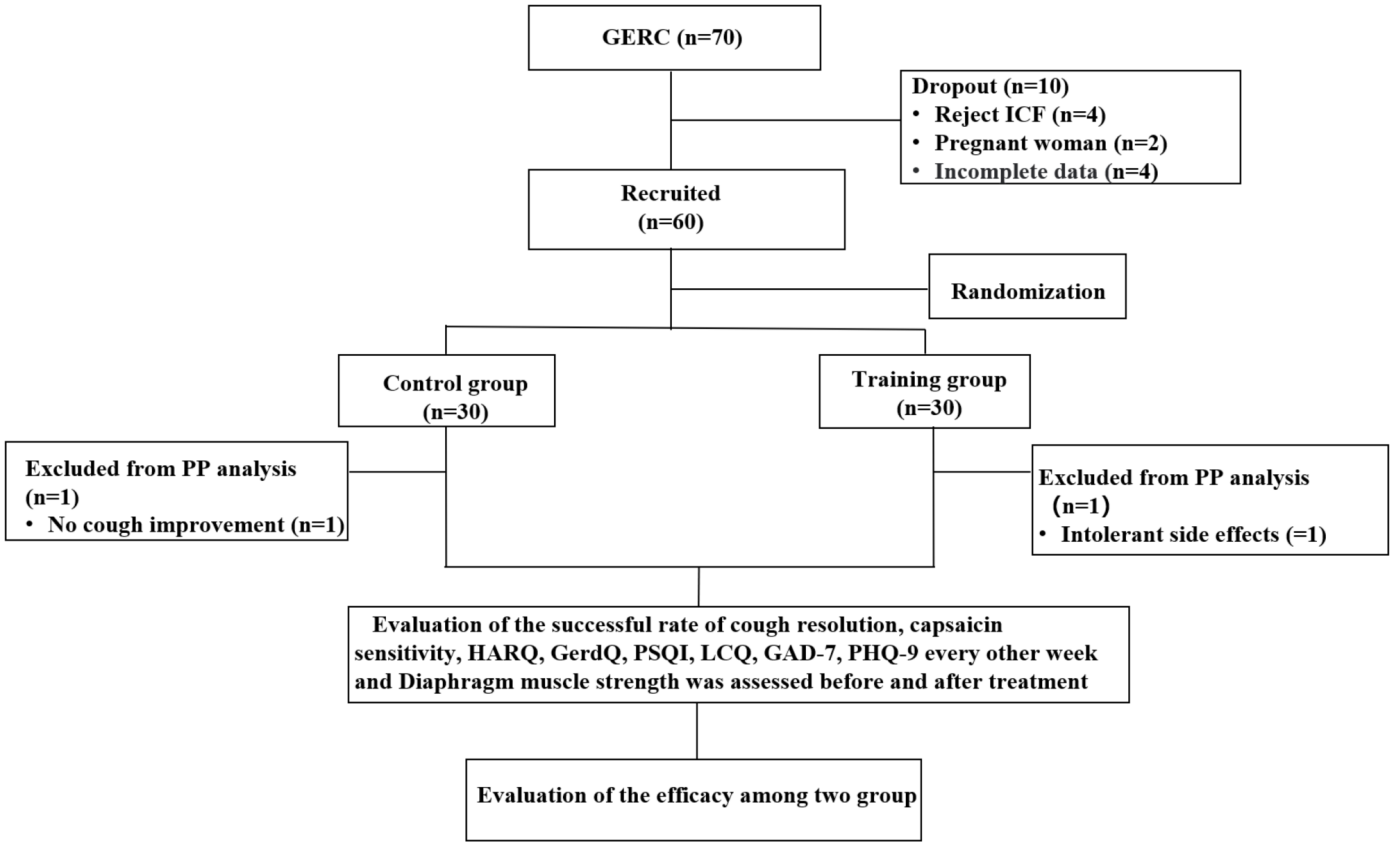
CONSORT (Consolidated Standards of Reporting Trials) flow diagram of the study. ICF: inform consent form; PP: pre-protocol; GERC: Gastroesophageal reflux-induced chronic cough; HARQ: Hull airway reflux questionnaire; GerdQ: Gastroesophageal reflux disease questionnaire; PSQI: Pittsburgh sleep quality index; LCQ: Leicester cough questionnaire; GAD-7: Generalized Anxiety Disorder Scale-7; PHQ-9: Patient Health Questionnaire-9

Before the enrollment and follow-up period, both groups received health education in the out-patient department: (a) avoid oversaturated bedtime eating, acid, spicy and greasy food, coffee, acid drinks and smoking; (b) head of the bed elevation and avoiding meals within 3 hours of bedtime. After enrollment, subjects were randomly divided into the intervention and the control group by computer-generated numbers. Patients were scheduled at separate times to receive individual attention and to avoid interparticipant contact. Moreover, to reduce the chance of bias emerging, team members separately acted as participant interviewers, data collators and evaluators to ensure all data were handled objectively. The cough symptom score, capsaicin cough sensitivity, Hull airway reflux questionnaire (HARQ), gastroesophageal reflux diagnostic questionnaire (GerdQ), generalized anxiety disorder scale-7 (GAD-7), patient health questionnaire-9 (PHQ-9), Pittsburgh sleep quality index (PSQI), the Leicester cough questionnaire (LCQ) was evaluated every two weeks for eight weeks. The changes in the above observation indexes at each time point in the two groups of patients were analyzed and the cough treatment effectiveness rate and the time difference of relief of each observation index were evaluated. Before and after treatment evaluated diaphragm muscle function by, diaphragm mobility, diaphragmatic thickening fraction measured by ultrasound and surface diaphragmatic EMG activity detected by surface electromyography (sEMG) were measured to compare the differences between the two groups and further evaluate the effect of DEP on the diaphragm. The consort flow diagram of study is shown in Fig. 1.

### Therapeutic regimen

Both groups were given standard anti-reflux treatment of omeprazole (AstraZeneca, China) 20 mg twice daily and mosapride (HaoSen, China) 5 mg three times daily, for eight weeks. If no remission of cough was achieved where the cough symptom score decreased by less than 50%, intensified anti-reflux treatment including increasing the dose of proton pump inhibitor (PPI) or adding a neuromodulator such as baclofen (Novartis, China) was given. In addition to this, the intervention group received professional training from a DEP rehabilitation trainer.

Briefly, when training before the study, the patient comfortably laid on the back and placed his hands on the abdomen to feel how the abdominal wall moves in and out. Repeat this exercise 5 to 10 times. Make sure that his breathing rhythm is calm and steady, and that the inflow and outflow of air feels natural. During each DEP session, the therapist tried to achieve good communication with the patient to facilitate good understanding and collaboration.

After the training, they performed independent training twice a day for 20 min each time, with a breathing frequency of six to eight breaths per minute for the eight-week trial period and specific training methods are provided in supplement 1. The patients were video-guided and were given a checklist on which they recorded whether they had undertaken training. Besides, their relations upload training videos for us. When the patient returned for a follow-up visit every two weeks, the rehabilitation trainer evaluated the patient’s progress and provided guidance for training.

### Outcome measures

The primary endpoint was the rate of cough resolution, as the sum of cough control and improvement. The cough was considered to be completely controlled when it disappeared, symptom score reduction of at least 50% was considered as cough improvement, and a cough symptom score reduction of less than 50%, no improvement, or aggravation was considered ineffective.

The second end-points included the changes in capsaicin cough sensitivity, HARQ, GerdQ, GAD-7, PHQ-9, PSQI, LCQ and diaphragm muscle performance.

### Auxiliary examination

For the capsaicin cough sensitivity test, based on the measurement method reported by Fujimura et al. [17], the modified method established in reference to the ERS guideline [18] was used. The minimum concentration of capsaicin required to induce > 2 (C2) or > 5 (C5) coughs as the subject’s cough threshold to evaluate the cough sensitivity to capsaicin.

The Chinese version of the cough symptom score [19], which evolved from the English version established by Hsu et al. [20] and verified clinically in the undergraduate department, was used to evaluate cough symptoms. Cough frequency and severity were divided into six levels, from zero for no cough to five for severe coughing most of the day. The Chinese version of the HARQ [21], which corresponds to the English version of the HARQ designed by Morice et al. [22], was used to assess cough hypersensitivity in patients. The GerdQ to assess reflux-related symptoms [23], was used to score reflux-related symptoms. The LCQ was used to evaluate the patient’s quality of life measure of chronic cough [24] and the PSQI was used to evaluate the patient’s sleep quality [25]. The GAD-7 [26] and PHQ-9 [27] were used to evaluate changes in patient anxiety and depressive moods.

To measure the diaphragm muscle function by ultrasound, a professional ultrasound technician used a Medison RS80A ultrasound machine (Samsung, South Korea) to measure the diaphragm mobility and thickening ratio. The measurement methods included diaphragm excursion (DE), where the subject was placed in a semi-recumbent position with the head of the bed elevated at 20 to 40° and a linear probe was placed at the intersection of the midline of the anterior chest wall and the costal arch to measure the right diaphragm through the liver as an acoustic window, scanning towards the head side. After identifying the diaphragm, the machine was switched to M-mode and the line perpendicular to the posterior one-third of the diaphragm was sampled. The distances from the baseline to the highest point during three respiratory cycles on the vertical axis were measured and averaged to obtain DE [28].

For the diaphragm thickening fraction (DTF), the patient was in the same position and the thickness of the diaphragm was measured at the intersection of the eighth to ninth intercostal space and the anterior axillary line and mid-axillary line at the end of inspiration and expiration [29]. The calculation for DTF was the difference in thickness between the end inspiration and the end of expiration divided by the thickness at the end-expiration × 100%.

Surface electrodes were used to assess EMG of the diaphragm muscle. All electromyography signals detected by the electrodes were transmitted to a biological signal acquisition and analysis system (ECH Probes, Shanghai) and amplified and band-pass filtered in the range of 5 Hz to 1 kHz, with a gain of 104 times. Under the condition of 2–6 kHz modulo sampling, the raw electromyography signals were converted into root mean square (RMS) time-domain and frequency-domain data using ECH probes electromyography acquisition and analysis software. The subjects performed a diaphragmatic maximal voluntary contraction (MVC) by performing the combined Mueller-expulsive maneuver with visual feedback and the data were normalized. The skin was lightly cleansed with alcohol to minimize electrical impedance, placing the recoding electrodes at the junction of the right sixth to eighth ribs and the anterior axillary line and the reference electrode at the bottom away from the recording electrode [30]. The electrode placement was recorded in about to 167 anatomical landmarks to ensure consistency in electrode placement between visits and as far as possible to avoid interference of intercostal muscles. The subject was placed in a semi-recumbent position with the head of the bed elevated at 20 to 40°, and observing the activity of diaphragmatic myoelectric signals to determine whether it was respiratory contraction. After the electromyography signal was free of artifacts, the sEMG of the diaphragm was continuously recorded during quiet breathing and abdominal deep breathing. From each recording 10 breaths free of artifacts were selected at the end of each period. The mean values were calculated after RMS smoothing processing and MVC normalized, respectively.

### Statistical analysis

According to previous studies [14], the effect size (d) for the two-tailed test was 0.80, the alpha value (a) was 0.05 and the statistical power (1-β) was 0.80. The sample size for the two groups was one-to-one, considering a dropout rate of 10%. Using G*Power 3.197, it was calculated that each group required 29 subjects and the total sample size was 58. To study the impact of outliers on the outcome, we used the Mahalanobis distance method to analyzed the two sets of results. By applying Mahalanobis distance method the outliers were refilled using the maximum value.

The primary efficacy analysis was evaluated using the modified intention-to-treat (ITT) method, which included all patients who received at least one dose of the study medication or a training session. All efficacy analyses were also assessed using the per-protocol (PP) method. Per-protocol population criteria included the following: subject received assigned study medication and DEP, was compliant with treatment.

For normally distributed data, the mean and standard deviation (SD) was used and for skewed distributed data, the median (Q1; Q3) were used. The cough threshold values C2 and C5 are logarithmically transformed and expressed as geometric mean for categorized data. The t-test, χ^2^ test, or Mann-Whitney U test were used to compare between-group and within-group differences. Statistical analysis was performed using the SPSS 24.0 software package (SPSS, USA). A P value less than 0.05 was considered statistically significant.

## Results

### General information

During the study period, a total of 70 GERC patients met the inclusion criteria. Ten patients were excluded due to exclusion criteria, including four patients who refused to sign the informed consent form, two pregnant women, four other patients with incomplete data. Sixty GERC patients were enrolled in the study, with 30 patients in the intervention group (56.7% of patients required additional treatment with neuromodulators) and 30 patients in the control group (53.3% of patients required additional treatment with neuromodulators). Adherence to DEP exercise training was achieved in 29 of 34 (85.3%), and taking medication in the control group was 29 of 32(90.6%). There was no statistical difference in adherence in each group (85.3% VS 90.6%; χ^2^ = 6.402, *P* = 0.507). There were no differences in the general clinical information and baseline observation indicators between the two groups, shown in Tables 1, 2 and 3. During the treatment period, one patient in the intervention group experienced persistent intolerable diarrhea after one week of treatment and refused to continue treatment. One patient in the control group did not show improvement in cough after three weeks and refused further treatment, so both were considered treatment failures.

**Table 1:** General clinical characteristics of patients

	Training group (*n* = 30)	Control group (*n* = 30)	Test results
Gender (M/F)	18/12	16/14	χ^2^ = 6.402, *P* = 0.602
Ages (years)	45.90 (13.19)	50.43 (15.35)	t=-1.227, *P* = 0.225
Cough duration (mo)	9.00 (2.75;12.50)	12.00 (3.00;21.00)	Z=-0.580, *P* = 0.562
Height (cm)	167.33 (7.20)	166.40 (9.38)	t = 0.432, *P* = 0.667
Weight (Kg)	64.83 (9.30)	67.83 (10.55)	t=-1.168, *P* = 0.247
BMI (Kg/m^2^)	23.10 (2.72)	24.53 (3.58)	t=-1.739, *P* = 0.087
Lung function (%)			
FEV1% pred	100.93 (13.16)	102.19 (16.07)	t = 0.263, *P* = 0.794
FVC% pred	100.70 (11.06)	100.45 (15.83)	t = 0.056, *P* = 0.956
FEV1/FVC%	84.58 (8.58)	86.12 (8.90)	t = 0.534, *P* = 0.597
Cough symptom score			
Daytime	3.00 (3.00;4.00)	3.00 (2.00;4.00)	Z=-1.063, *P* = 0.288
Nighttime	2.00 (1.00;2.00)	1.00 (1.00;2.00)	Z=-0.621, *P* = 0.535
Capsaicin cough threshold			
C2 (µmol/L)	0.86 (0.05)	0.88 (0.08)	t=-0.896, *P* = 0.374
C5 (µmol/L)	0.93 (0.18)	0.94 (0.17)	t=-0.221, *P* = 0.826

Data are presented as mean (SD), median (Q1; Q3)

FEV1, forced expiratory volume in one second; FVC, forced vital capacity; C2, capsaicin solution concentration with ≥ 2 coughs; C5, capsaicin solution concentration for ≥ 5 coughs

**Table 2:** Comparison of variables of MII-pH between two groups

	Training group (*n* = 30)	Control group (*n* = 30)	Test results
DeMeester score	16.64 (3.38;24.05)	13.39 (4.64;23.06)	Z=-0.621, *P* = 0.535
AET (%)	5.61 (0.90;8.03)	5.84 (2.83;6.98)	Z=-0.200, *P* = 0.842
Acid SAP (%)	96.65 (18.60;99.53)	97.80 (19.48;100.00)	Z=-0.736, *P* = 0.464
Non-acid SAP (%)	22.70 (0.00;99.80)	21.35 (5.23;99.80)	Z=-0.703, *P* = 0.482
SI (%)	59.70 (28.35;79.48)	63.15 (31.50;87.68)	Z=-0.421, *P* = 0.673
Acidic reflux (n)	71.00 (17.25;95.25)	76.50 (24.75;108.25)	Z=-0.326, *P* = 0.717
Weakly acidic reflux (n)	40.00 (9.75;82.50)	29.00 (12.00;72.75)	Z=-0.015, *P* = 0.988
Weakly alkaline reflux (n)	6.50 (3.00;24.00)	9.50 (2.75;24.00)	Z-0.318, *P* = 0.750
Gas reflux (n)	37.50 (19.25;61.50)	42.00 (19.75;71.00)	Z=-0.422, *P* = 0.673
Liquid reflux (n)	48.50 (18.50;77.00)	44.00 (28.50;64.75)	Z=-0.303, *P* = 0.762
Mixed reflux (n)	33.00 (12.75;56.25)	36.00 (19.75;67.50)	Z=-0.673, *P* = 0.501
Proximal extent (n)	11.00 (7.75;22.00)	14.50 (6.00;22.00)	Z=-0.600, *P* = 0.549
Total number of reflux episodes (n)	110.50 (97.25;207.25)	131.50 (101.50;157.75)	Z=-0.924, *P* = 0.355

Data are presented as median (Q1; Q3)

AET, acid exposure time; SAP, symptom association probability; SI, symptom index; n, number of times. DeMeester score was automatically reported by Database software as a global measure of esophageal acid exposure. Proximal extent was defined as the number of reflux events reaching ≥ 15 cm above the lower esophageal sphincter

**Table 3:** Comparison of variables between two groups

	Training group (*n* = 30)	Control group (*n* = 30)	Test results
GerdQ	7.93 (1.72)	8.23 (2.03)	t=-0.618, *P* = 0.539
LCQ	13.55 (2.66)	13.59 (2.41)	t=-0.069, *P* = 0.946
GAD-7	3.00 (2.00;8.25)	3.00 (0.00;4.25)	Z=-1.038, *P* = 0.299
PHQ-9	2.00 (0.75;4.00)	2.00 (0.00;4.25)	Z=-0.060, *P* = 0.952
PSQI	7.00 (4.00;9.25)	6.00 (4.75;7.25)	Z=-0.900, *P* = 0.368
HARQ	21.90 (8.43)	22.23 (9.96)	t=-0.140, *P* = 0.889

Data are presented as mean (SD) or median (Q1; Q3)

GerdQ, Gastroesophageal reflux disease questionnaire; LCQ, Leicester cough questionnaire; GAD-7, Generalized Anxiety Disorder Scale-7; PHQ-9, Patient Health Questionnaire-9; PSQI, Pittsburgh sleep quality index; HARQ, Hull airway reflux questionnaire

### Comparison of cough resolution rate between the two group

**Fig. 2:**
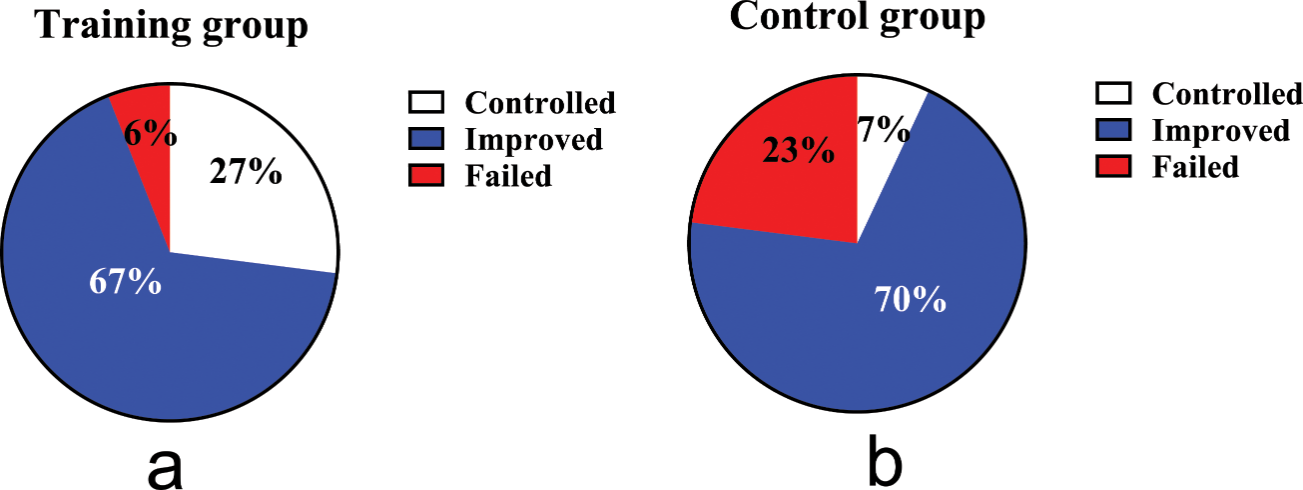
Comparison of therapeutic outcomes. (**a**): the cough treatment efficacy of the training group; (**b**): the cough treatment efficacy of the control group. The rate of cough resolution in the training group is significantly higher than in the control group (94% VS 77%, *P* = 0.041 by ITT, *P* = 0.027 by PP analysis)

A total of 58 out of 60 GERC patients (97%) completed the study. After eight weeks of treatment, by the ITT analysis, the cough treatment efficacy in the intervention group of 94% was significantly higher than that in the control group at 77% (χ^2^ = 6.402, *P* = 0.041), as same as the PP analysis (χ^2^ = 7.196, *P* = 0.027), as shown in Fig. 2.

### Comparison of scales evaluation and capsaicin cough sensitivity before and after treatment

After eight weeks of treatment, according to ITT analysis, the intervention group showed more significant improvements than the control group in terms of nighttime cough symptoms score (Z = -2.027, *P* = 0.043), GerdQ (t = -2.800, *P* = 0.007), GAD-7 (Z = -2.096, *P* = 0.036), PSQI (Z = -3.705, *P* < 0.000), LCQ (t = 2.911, *P* = 0.005) and PHQ-9 (Z = -2.111, *P* = 0.035), while there was no statistically significant difference in capsaicin cough sensitivity (C2: t = 0.685, *P* = 0.496; C5: t = 1.070, *P* = 0.289) and HARQ (t = -1.754, *P* = 0.085) between the two groups. The intervention group showed faster relief of the nighttime cough symptoms score than the control group in the fourth week (Z = -2.667, *P* = 0.007), and.

**Fig. 3:**
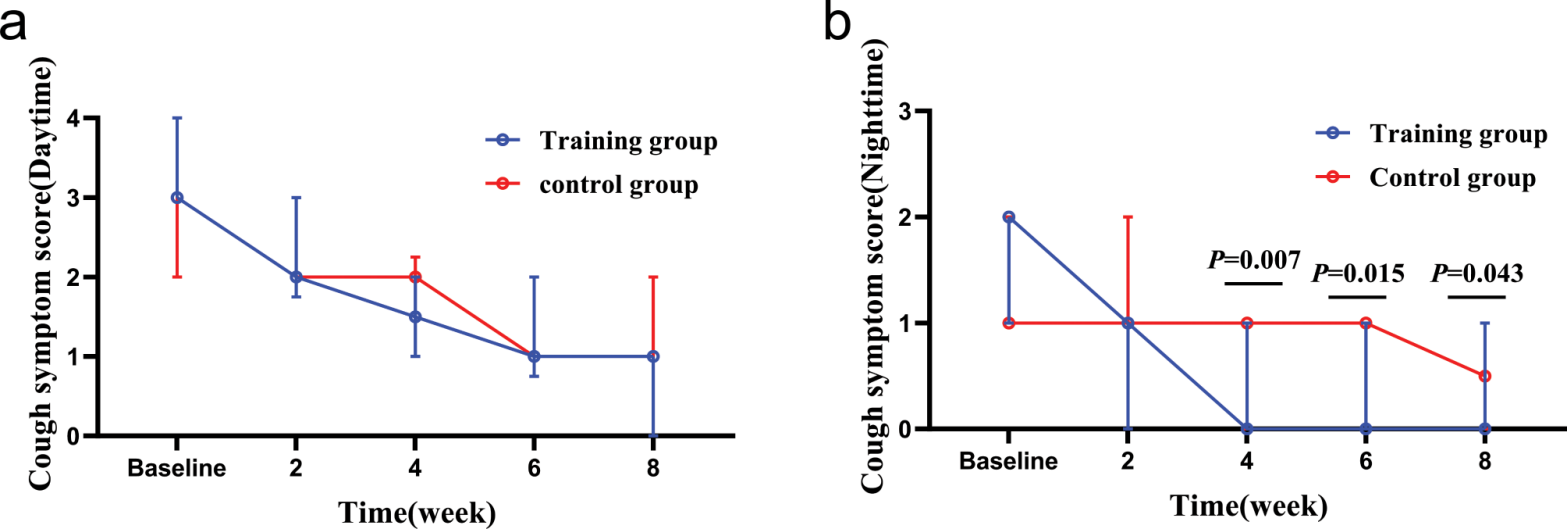
Changes in cough symptom score from baseline to the 8-week treatment between the two groups. (**a**): changes in daytime cough symptom score over time; (**b**): changes in nighttime cough symptom score over time. In the fourth week, the training group than the control group obviously relieve nighttime cough symptoms

**Fig. 4:**
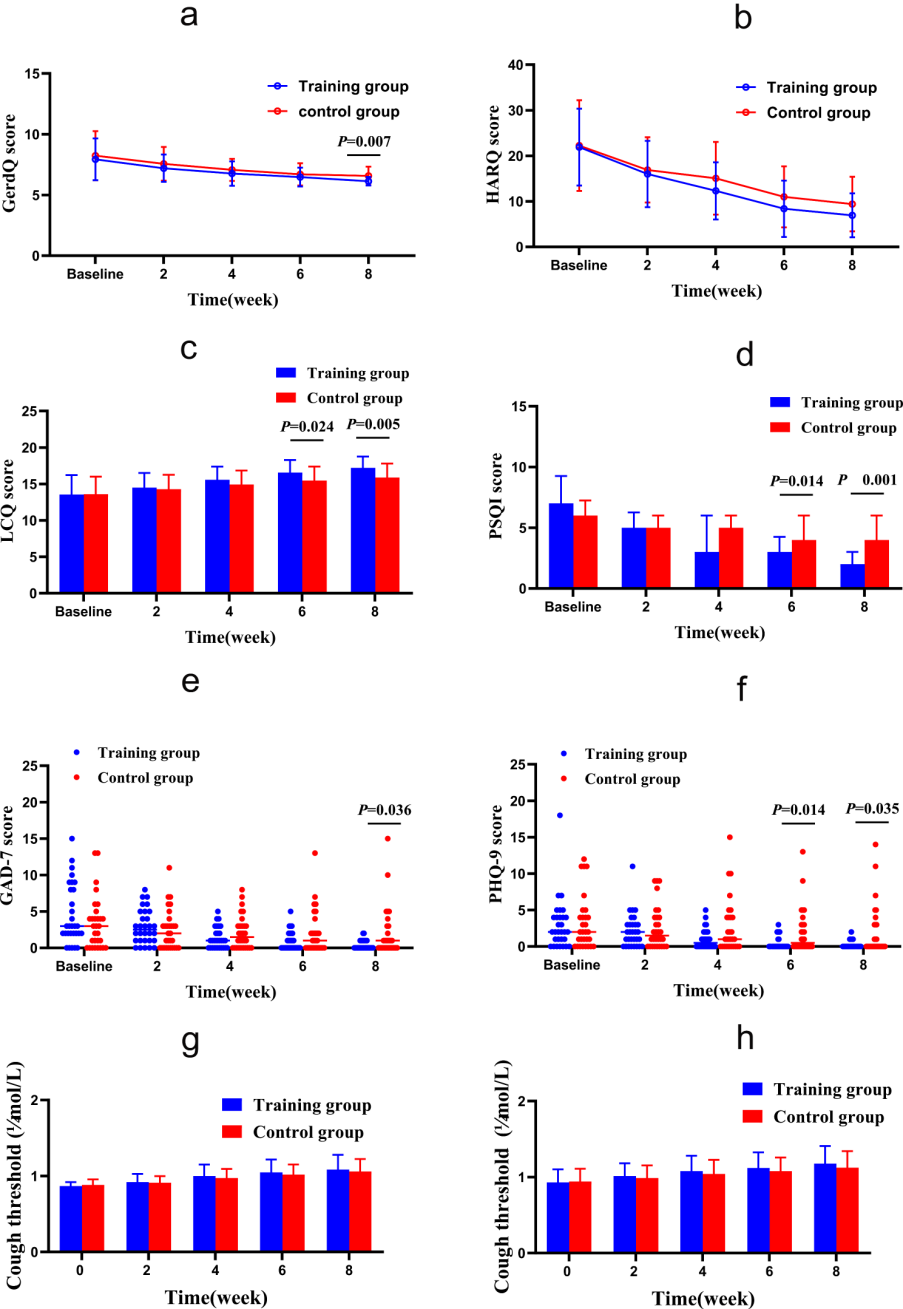
Changes of GerdQ, HARQ, LCQ, PSQI, GAD-7 and PHQ-9 from baseline to the 8-week treatment in the two groups. (**a**) GERC: Gastroesophageal reflux-induced chronic cough; (**b**) HARQ: Hull airway reflux questionnaire; (**c**) LCQ: Leicester cough questionnaire; (**d**) PSQI: Pittsburgh sleep quality index; (**e**) GAD-7: Generalized Anxiety Disorder Scale-7; (**f**) PHQ-9: Patient Health Questionnaire-9; (**g**) Capsaicin cough sensitivity: cough threshold C2; (**h**) Capsaicin cough sensitivity: cough threshold C5. *: *P*<0.05. After 8 weeks of treatment, GerdQ, LCQ, PSQI, GAD-7 and PHQ-9 in the intervention group were significantly relieved compared with those in the control group. In addition, LCQ, PSQI and PHQ-9 alleviated faster

LCQ, PHQ-9 and PSQI improved faster in the sixth week than the control group, as shown in Figs. 3 and 4.

There are also significant difference in the improvement of nighttime cough symptoms score, GerdQ, GAD-7, PSQI, LCQ and PHQ-9 in the intervention group was noted on PP analysis.

### Comparison of DE, DTF and sEMGdi between the two groups

**Fig. 5:**
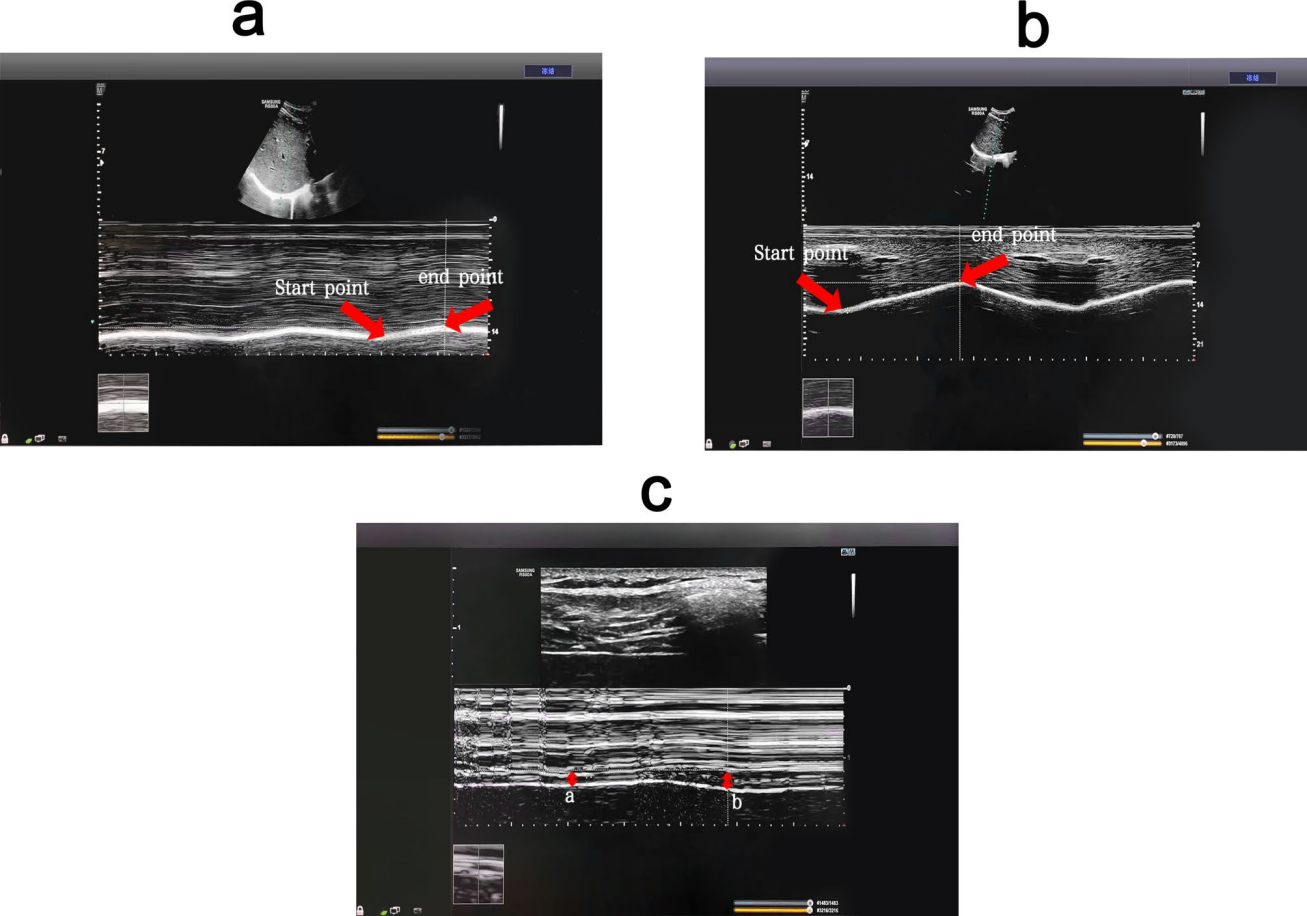
(Pre-treatment) Comparison of diaphragm excursion and diaphragm thickening fraction among breathing types. (**a**) Changes of diaphragm excursion at quiet breathing. (**b**) Changes of diaphragm excursion at abdominal deep breathing. (**c**) Changes of diaphragm thickness (**a**: changes of diaphragm thickness at quiet breathing; **b**: changes of diaphragm thickness at abdominal deep breathing). DEP can significantly increase DE and DTF compared with quiet breathing. DEP, deep diaphragmatic breathing training; DE, diaphragmatic excursion; DTF, diaphragm thickening fraction

**Fig. 6:**
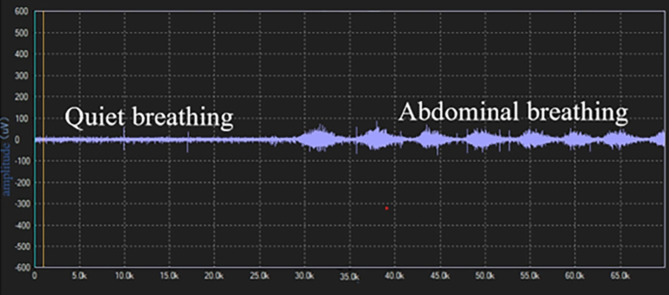
(Pre-treatment) Comparison of diaphragm EMG activity among breathing types Diaphragm sEMG activity was higher during abdominal than quiet breathing. sEMG: surface electromyogram activity

Before treatment, there was no significant difference in baseline data between the 22 patients in the intervention group and 20 in the control group, who completed the diaphragm examination (*P* > 0.05) Supplementary Table 1. The diaphragm mobility, diaphragm thickening rate and diaphragm sEMG activity of both groups during DEP were significantly higher than during quiet breathing, shown in Figs. 5 and 6. Before treatment, the sEMGdi (training group: t = 7.808, *P*<0.001; control group: t = 8.172, *P*<0.001) during DEP has statistically significant contrast with quiet breathing which was consistent with DE (training group: t = 39.773, *P*<0.001; control group: t = 33.261, *P*<0.001) and DTF (training group: t = 17.970, *P*<0.001; control group: t = 14.620, *P*<0.001).

After eight weeks of treatment, the sEMGdi of the intervention group was significantly higher than that of the control group during DEP (t = 6.288, *P* <0.001) and quiet breathing (t = 8.136, *P* <0.001). The DTF of the intervention group at 169.50 (22.47) was significantly higher than that of the control group during DEP at 150.55 (25.54) (t = 2.558, *P* = 0.014). The measurement of DE showed that there was no statistically significant difference in diaphragm mobility between the two groups of both quiet breathing and DEP (*P* > 0.05). (Table 4.)

**Table 4:** Comparison of DE, DTF and EMG of between the two groups (post-therapy)

	Abdominal Breathing			Quite Breathing		
	GERC group (n = 22)	Control group (n = 20)	Test results	GERC group (n = 22)	Control group (n = 20)	Test results
DE (dm)	0.51 (0.05)	0.51 (0.04)	t = 0.121, *P* = 0.904	0.17 (0.02)	0.17 (0.00)	t = 0.116, *P* = 0.908
DTF (%)	169.50 (22.47)	150.55 (25.54)	t = 2.558, *P* = 0.014	59.82 (11.08)	55.10 (12.85)	t = 1.278, *P* = 0.209
sEMdi (%MVC)	79.00 (2.49)	74.65 (1.93)	t = 6.228, *P* = 0.000	72.73 (1.96)	67.15 (2.48)	t = 8.136, *P* = 0.000

Data are presented as mean (SD)

DE: Diaphragm excursion; DTF: Diaphragm Thickening fraction; sEMGdi: surface diaphragmatic EMG activity; MVC: maximal voluntary contraction

### Comparison of DE, DTF and sEMGdi before and after treatment

In the intervention group, the post-treatment sEMG activity of the diaphragm muscle during both DEP and quiet breathing increased significantly compared to pre-treatment (*P* < 0.05). The B-ultrasound measurement of diaphragm mobility during DEP of post-treatment increased significantly compared to pre-treatment(*P* < 0.05), while there was no statistically significant difference during quiet breathing (*P* > 0.05). The DTF during both DEP and the quiet breathing of post-treatment increased compared to pre-treatment, but there was no statistically significant difference (*P* > 0.05) Supplementary Table 2. In the control group, there were no significant statistical differences observed in the post-treatment of the sEMG of the diaphragm in DEP (*P* > 0.05) and quiet breathing (*P* > 0.05) compared to pre-treatment. There were also no statistical differences in diaphragmatic excursion and DTF before and after treatment as well. Supplementary Table 3.

## Discussion

The present study found that compared to single anti-reflux medication therapy, the combination of DEP can improve the effectiveness of GERC treatment. Compared to the control group, the intervention group showed more significant improvements in the overall evaluation of GerdQ, LCQ, PSQI, GAD-7 and PHQ-9.

The presence of GERC is an important extraesophageal manifestation of GERD. According to the pathogenesis of GERD, the weakening of the anti-reflux barrier function plays an important role in the occurrence and development of GERC. The LES and diaphragm are important components of the anti-reflux barrier. The LES is a circular muscle layer at the distal end of the esophagus. Its resting pressure is usually sufficient to prevent gastric contents from refluxing into the esophagus. However, when abdominal pressure increases, the diaphragm forms a second defense barrier to prevent reflux [31]. When LES is surgically removed, pressure can still be detected at the gastroesophageal junction [32], indicating that the diaphragm continues to maintain the anti-reflux barrier function, emphasizing the important role of the diaphragm in the anti-reflux barrier. Several studies have shown that respiratory training can increase diaphragm function [33, 34]. The DEP technique mainly completes deep, slow and regular breathing through diaphragm contraction and relaxation. Eherer et al. found that DEP reduced acid reflux exposure in GERD patients, improved reflux symptoms and speculated that DEP training can train the crural diaphragm and reinforce the anti-reflux barrier [14].

Studies have also shown that most reflux events in GERD occur during periods of transient lower esophageal sphincter relaxation (TLESR) [35]. In addition to LES relaxation, the inhibition of the diaphragm muscle is an essential part of TLESR occurrence [31]. Banovcin et al. found that acid stimulation of the esophageal nerves can enhance gastric distension and cause a TLESR reflex, possibly by acid-activating sensory nerves in the esophagus and increasing the frequency of TLESR [36]. The use of PPIs can alleviate acid exposure-induced TLESR to some extent but cannot reduce reflux caused by LES and diaphragm dysfunction or decrease the frequency of reflux. Coughing caused by reflux is related to the total amount of proximal reflux and prolonged esophageal reflux exposure, rather than the pH value of the reflux, so most patients cannot benefit from acid suppression therapy [37].

Halland et al. found that DEP training can significantly reduce the frequency of reflux and decrease postprandial acid exposure, further improving cough symptoms in GERD [38].

Previous studies have indicated that both TLESR and the diaphragm muscle are regulated by the vagus nerve [39]. The nerve regulator baclofen is a γ-aminobutyric acid (GABA) receptor agonist that can regulate the vagus nerve pathway, reduce the occurrence of TLESR and decrease the frequency of reflux, thereby relieving cough symptoms in GERD, which is applied clinically [40]. However, some patients cannot tolerate baclofen due to the central nervous system side effects such as dizziness, drowsiness and fatigue [41]. The use of DEP training can directly or indirectly regulate the balance between sympathetic and parasympathetic nerves and is used in GERD, anxiety and other diseases [12, 14, 42]. Perhaps through the above mechanism, it can indirectly reduce the occurrence of TLESR, improve diaphragm function, reduce the use of baclofen and increase patient compliance with treatment.

Currently, the treatment for GERC includes medication, surgery, as well as non-pharmacological and non-surgical intervention. As people’s quality of life demands continue to rise, physical exercise and lifestyle modifications interventions for GERC are increasingly important. The guideline also points out that for suspected GERC patients without symptoms of acid reflux or heartburn, PPIs should not be the first choice and lifestyle and behavioral interventions should be prioritized [43]. Although non-pharmacological or lifestyle modifications interventions have been widely recommended for GERD patients in recent years, they are rarely mentioned for GERC patients. To the best of our knowledge, this study is the first clinical randomized controlled study on deep diaphragmatic breathing training interventions for GERC and it was concluded that this type of intervention could significantly improve the clinical symptoms of GERC patients in conjunction with medication therapy.

Based on the above mechanisms and research results, it is hypothesized that DEP training can improve the clinical symptoms of GERC patients by improving diaphragm muscle function, strengthening the anti-reflux barrier, regulating the vagal reflex, reducing the occurrence of TLESR.

To further confirm the mechanism of DEP training on the diaphragm, this study objectively evaluated diaphragm function through multiple methods. Transdiaphragmatic pressure is the main indicator for evaluating diaphragm contraction function [44], but it is invasive and difficult to widely implement in clinical practice. In recent years, studies have shown that diaphragm ultrasound can indirectly evaluate diaphragm contraction force assessing DE and DTF [45]. DE and DTF had be used to evaluate diaphragmatic function and predicted weaning from mechanical ventilation in many researches. To our knowledge, the usefulness of this technique in evaluating the changes in diaphragm function before and after DEP and speculating the effect of respiratory training on GERC has not been reported. The results showed that during DEP, diaphragm mobility was significantly increased compared to calm breathing, indicating that the diaphragm function increased accordingly, consistent with the results of Yamaguti et al. [13], and the DTF was significantly increased at post-treatment contrast to control group, indicating that DEP effectively trains the diaphragm. Compared with Wu W, et al. research on diaphragm mobility before and after rehabilitation [30], the change value did not change much and the ultrasonic sampling will be subject to echo error, for which the possibility of error cannot be excluded. The clinical significance of DEP needs to be further confirmed by large sample and multi-center independent studies. Moreover, the cause-and-effect relationship between the changes in the diaphragm and cough has not been established. Therefore, further research is necessary.

The sEMG can also quantify the work of respiratory muscles and serve as a non-invasive method to indirectly reflect respiratory muscle function [46]. In this study, sEMG was used to measure the diaphragm electromyographic activity of patients during DEP and calm breathing to evaluate changes in diaphragm contraction force. After 8 weeks of treatment, the diaphragm sEMG activity in the training group was increased in quiet breathing and deep abdominal breathing compared with those before training, in line with DE and DTF, indicating that the diaphragm function was improved under DEP. In the control group, the diaphragm electromyography activity showed an increasing trend at quiet breathing and a decreasing trend at abdominal deep breathing. The DE and DTF were not significantly or slightly increased. It may reflect that the diaphragm is prone to fatigue and its function has not improved and may be gradually deteriorating. Cough symptoms may reappear after drug withdrawal, which needs further study. The contamination of the signal picked up by surface electrodes aimed at recording diaphragm activity has also been reported. But, Similowski, et al., and Verin E, et al. [47, 48] found that when two recording EMG electrodes are placed very close to one another, they are much more likely to record near-field potentials than far-field potentials. And the surface electrodes could be silent in response to cervical magnetic stimulation in patients with phrenic paralysis. Therefore, we believe that, surface electrodes may provide an uncontaminated diaphragm signal. And we will further to study the correlation of sEMG, di with EMGdi.

GERC is a special type of GERD manifested by a prominent cough symptom. Eherer et al. [14] research demonstrated that diaphragmatic breathing significantly reduced acid exposure and improved symptoms of GERD. Compared to the research, the patients in our study had a much wider age range, were fatter, and the standard of living was higher, leading to more difficulty in curing. Our study showed that the intervention group showed significant improvement in their gastroesophageal reflux symptoms and quality of life compared to the control group, in line with Eherer et al. research. However, the cough symptoms relief was faster than gastroesophageal reflux symptoms. Some research showed that GREC pathogenesis mainly includes two theories: reflux theory and reflex theory. DEP may not only improve diaphragmatic function, but also is significantly associated with increased thalamic GABA levels and reduced sensitivity of the cough center. The pathogenesis of GERD is complex and the prime is reflux exposure, so it is slower to relieve than cough symptoms.

In recent years, the incidence of GERC has been increasing due to changes in people’s lifestyles, improvements in corresponding diagnostic techniques and increased awareness of the disease, which is making an increasingly significant impact on people’s quality of life [5]. The LCQ, GAD-7 and PHQ-9 can measure the quality of patients’ lives. Comparing GAD-7 and PHQ-9, LCQ can comprehensively evaluate the impact of cough on patients’ lives from the physiological, psychological and social aspects. This study used the LCQ score to comprehensively evaluate changes in patients’ quality of life and found that patients who underwent DEP training were able to improve their quality of life more quickly, strengthening their treatment compliance. For chronic cough patients, especially during the pandemic, long- term uncontrollable coughing can lead to anxiety and depression, and frequent nighttime coughing can affect sleep quality, exacerbating emotional disorders. Psychological disorders can worsen patients’ sensitivity to symptoms and reduce their treatment compliance and GERD patients are more prone to comorbid anxiety and depression, leading to treatment difficulties [10, 49] and a detrimental cycle. The DEP training is a relaxation technique that may upregulate GABA [50], regulate the balance of the sympathetic and parasympathetic nervous systems, reduce cortisol secretion, lower respiratory rate and increase heart rate variability, relieving patients’ anxiety and other emotions [13, 51] and reducing symptom sensitivity caused by these disorders. Gu et al. found that DEP training improved patients’ psychiatric disorders and improved sleep quality by reducing negative emotions [42]. The changes in cough symptoms, anxiety and depression and sleep quality in the intervention group in this study were consistent with the above research results, further supporting the benefits of DEP training for GERC.

Gabapentin, a widely used neural regulator in clinical practice, is a GABA derivative that inhibits synaptic neurotransmitter release, thereby inhibiting the sensitivity of the cough center to reduce coughing [7]. Previous studies in this department have found that gabapentin is effective for refractory GERC, possibly because these patients have cough center hypersensitization [52] and Streeter C, et al. found that breathing was significantly associated with increased thalamic GABA levels using magnetic resonance spectroscopy [50]. , which may be another mechanism for alleviating coughing in GERC patients.

HARQ and capsaicin cough sensitivity test were related to cough hypersensitivity. In this study, the HARQ and capsaicin cough sensitivity test showed an improvement trend after 8 weeks of training while these values showed no statistically significant difference(Supplementary Table 4), which further confirms the DEP may inhibit the sensitivity of the cough center and relieve cough symptoms in patients with GERC.

This study had some limitations. (1) In view of the pain of the examination, patients did not want to repeat the examination, especially after the symptoms improved, so we did not require the acquisition of esophageal manometry and MII-PH data in the design of the study protocol. While the improvement in diaphragmatic muscle function was observed through B-mode ultrasound and sEMG, the changes in pressure at the gastroesophageal junction and acid exposure could not be obtained. The direct relationship between diaphragmatic muscle strength enhancement and reflux cannot therefore be confirmed. (2) The ultrasonic sampling will be subject to echo error, for which the possibility of error cannot be excluded. (3) The sample size of this study is also relatively small, mainly because the proportion of these GERC patients was very low, and it is difficult for some patients to persist in training DEP, and larger studies may be needed to support the conclusions.

## Conclusions

The DEP training may increase patients’ diaphragmatic muscle function, therefore, enhance anti-reflux barriers, improve cough treatment effectiveness in patients with GERC and alleviate symptoms of gastroesophageal reflux, improve quality of life, sleep quality and alleviate anxiety and depression.

### Electronic supplementary material

Below is the link to the electronic supplementary material.

Supplementary Material 1

Supplementary Material 2

Supplementary Material 3

## Data Availability

Some or all datasets generated during and/or analyzed during the current study are not publicly available but are available from the corresponding author on reasonable request.

## Abbreviations

AET: acid exposure time
DE: diaphragmatic excursion
DEP: deep diaphragmatic breathing training
DTF: diaphragm thickening fraction
FEV1: forced expiratory volume in one second
FVC: forced vital capacity
GABA: γ-aminobutyric acid
GAD-7: Generalized Anxiety DisorderScale-7
GERC: gastroesophageal reflux-induced chronic cough
GERD: gastroesophageal reflux disease
GerdQ: Gastroesophageal reflux diagnostic questionnaire
HARQ: Hull airway reflux questionnaire
ICF: inform consent form
ITT: intention-to-treat
LCQ: Leicester cough questionnaire
LES: lower esophageal sphincter
MII-pH: Multichannel intraluminal esophageal impedance and pH monitoring
MVC: maximal voluntary contraction
PHQ-9: Patient Health Questionnaire-9
PP: per-protocol
PPI: proton pump inhibitor
PSQI: Pittsburgh sleep quality index
RMS: root mean square
SAP: symptom association probability
sEMG: surface electromyogram activity
sEMGdi: surface diaphragmatic EMG activity
SI: symptom index
TLESR: transient lower esophageal sphincter relaxation
