# Supplementary material for: Positive effect of deep diaphragmatic breathing training on gastroesophageal reflux-induced chronic cough: a clinical randomized controlled study
Source: Respir Res. 2024 Apr 18;25:169. doi: 10.1186/s12931-024-02783-5 (PMC11027235; doi:10.1186/s12931-024-02783-5)
Supplement: Supplementary file 1 — Supplementary Material 1 [file 12931_2024_2783_MOESM1_ESM.doc]

# Breathing Training

# Preface

The training program outlined in this brochure was compiled for a study conceived and directed by Andreas Eherer (Associate Professor of Medicine, Medical University of Graz) and aimed at improving gastroesophageal reflux-related symptoms.

The exercises are based on a training program developed by Karl Ernst Hoffmann (Professor Emeritus of Voice at the University of Music and Dramatic Arts, Graz) for his students.

The exercises were adapted by Gernot Puschnig (student at the Academy of Physiotherapy, Graz) to make them more suitable for alleviating gastroesophageal reflux.

According to this, we modified it slightly so that it fits better our project.

# General remarks on the training program

Read this brochure carefully before you start training.

You will need about 20 to 30 minutes for whole set of exercises(a total of 8 weeks, 2 times a day ). Make sure you have enough time for your training session. The training will be effective only if you feel calm and relaxed while you are doing the exercises!

Every exercise should be continued until the accompanying music stops.

If you start to feel dizzy or faint during your training session, stop the exercise, relax and breathe normally. Continue with the exercises once your discomfort has passed.

If possible, always do the exercises at the same time of the day, preferably before the evening meal or at least one hour thereafter.

Note whether your symptoms improve in the course of the training and record any changes in your training protocol.

It is important that you record in your training protocol your all medications taken during the training period.

# Description of the starting positions

**Starting position: lying on back (supine position)**

You should best lie on the floor on a gymnastics mat, a soft blanket or a carpet. Bend your legs to avoid excessive curving of your lumbar spine (swayback). Put a pillow under your head. Hold your arms at your sides, straight but relaxed.

**Exercise**

Starting position: supine

Put your left hand on your breastbone, your right hand on your navel. Breathe in through your nose as if trying to draw in a pleasant aroma. Image you want to inhale a beautiful fragrance as deeply as possible in one breath.

Make sure that only your stomach rises when you breathe in. With your left hand, make sure that your thorax remains entirely relaxed, while your right hand rises and falls with the rhythm of your breathing. Breathe out through your nose. With your right hand you should be able to feel how the abdominal wall moves in and out.

Repeat this exercise 5 to 10 times; then change the position of your hands.

Make sure that your breathing rhythm is calm and steady, and the inflow and outflow of air feels natural.
