# Supplementary material for: Positive effect of deep diaphragmatic breathing training on gastroesophageal reflux-induced chronic cough: a clinical randomized controlled study
Source: Respir Res. 2024 Apr 18;25:169. doi: 10.1186/s12931-024-02783-5 (PMC11027235; doi:10.1186/s12931-024-02783-5)
Supplement: Supplementary file 3 — Supplementary Material 3 [file 12931_2024_2783_MOESM3_ESM.docx]

**Table 1.** Comparison of DE, DTF and sEMGdi between the two groups (pre-treatment)

|  | | Abdominal Breathing | | | | |  | | | | | Quite Breathing | | | | | |  |
| --- | --- | --- | --- | --- | --- | --- | --- | --- | --- | --- | --- | --- | --- | --- | --- | --- | --- | --- |
|  |  | GERC group (n=22) | | Control group (n=20) | Test results | | |  | | | GERC group (n=22) | | | Control group (n=20) | | Test results |  |  |
| DE (dm) | 0.49 (0.04) | | 0.50 (0.04) | | | t=-0.272, *P*=0.787 | | |  | 0.16 (0.02) | | | 0.17 (0.01) | | t=-1.299, *P*=0.201 | | | |
| DTF (%) | 159.73 (30.90) | | 146.30 (29.23) | | | t=1.443, *P*=0.157 | | |  | 56.95 (11.61) | | | 51.30 (10.19) | | t=1.670, *P*=0.103 | | | |
| sEMGdi, | 76.27 (4.78) | | 74.75 (4.08) | | | t=1.105, *P*=0.276 | | |  | 65.95 (3.48) | | | 65.20 (3.14) | | t=0.735, *P*=0.467 | | | |
| ( %MVC) |  | |  | | |  | | |  |  | | |  | |  | | | |

Data are presented as mean (SD)

DE: Diaphragm excursion; DTF: Diaphragm Thickening fraction; sEMGdi: surface diaphragmatic EMG activity;

**Table 2.** Changes of DE, DTF and sEMG in different breathing types after 8 weeks of treatment in training group

|  | | Abdominal Breathing | | | | |  | | | | | Quite Breathing | | | | |  |  |
| --- | --- | --- | --- | --- | --- | --- | --- | --- | --- | --- | --- | --- | --- | --- | --- | --- | --- | --- |
|  |  | GERC group (n=22) | | Control group (n=20) | Test results | | |  | | | GERC group (n=22) | | | Control group (n=20) | | Test results | |  |
| DE (dm) | 0.49 (0.04) | | 0.51 (0.05) | | | t=-3.039, *P*=0.006 | | |  | 0.16 (0.02) | | | 0.17 (0.02) | | t=-0.310, *P*=0.760 | | | |
| DTF (%) | 159.72 (30.90) | | 169.50 (22.47) | | | t=-2.072, *P*=0.051 | | |  | 56.95 (11.61) | | | 59.82 (11.08) | | t=-1.665, *P*=0.111 | | | |
| sEMGdi, | 76.27 (4.78) | | 79.00(2.49) | | | t=-2.494, *P*=0.021 | | |  | 65.95 (3.48) | | | 72.73 (1.96) | | t=-7.653, *P*=0.000 | | | |
| (%MVC) |  | |  | | |  | | |  |  | | |  | |  | | | |

Data are presented as mean (SD)

DE: Diaphragm excursion; DTF: Diaphragm Thickening fraction; sEMGdi: surface diaphragmatic EMG activity

**Table 3.** Changes of DE, DTF and sEMG in different breathing types after 8 weeks of treatment in control group

|  | | Abdominal Breathing | | | | |  | | | | | Quite Breathing | | | | |  |  |
| --- | --- | --- | --- | --- | --- | --- | --- | --- | --- | --- | --- | --- | --- | --- | --- | --- | --- | --- |
|  |  | GERC group (n=22) | | Control group (n=20) | Test results | | |  | | | GERC group (n=22) | | | Control group (n=20) | | Test results | |  |
| DE (dm) | 0.50 (0.04) | | 0.51 (0.04) | | | t=-1.060, *P*=0.302 | | |  | 0.17 (0.01) | | | 0.17 (0.00) | | t=1.990, *P*=0.061 | | | |
| DTF (%) | 146.30 (29.23) | | 150.55 (25.54) | | | t=-1.737, *P*=0.099 | | |  | 51.30 (10.19) | | | 55.10 (12.85) | | t=-1.158, *P*=0.261 | | | |
| sEMGdi, | 74.75(4.08) | | 74.65 (1.93) | | | t=0.090, *P*=0.930 | | |  | 65.20(3.14) | | | 67.15 (2.48) | | t=-2.012, *P*=0.059 | | | |
| (%MVC) |  | |  | | |  | | |  |  | | |  | |  | | | |

Data are presented as mean (SD)

DE: Diaphragm excursion; DTF: Diaphragm Thickening fraction; sEMGdi: surface diaphragmatic EMG activity

**Table 4.** Comparison of variables between two groups. (post-treatment)

|  | Training group  (n=30) | Control group  (n=30) | Test results |
| --- | --- | --- | --- |
| GerdQ | 6.13 (0.34) | 6.57 (0.77) | t=-2.800, *P*=0.007 |
| LCQ | 17.19 (1.56) | 15.88 (1.92) | t=2.911, *P*=0.005 |
| GAD-7 | 0.00 (0.00;1.00) | 1.00 (0.00;3.00) | Z = -2.096, P = 0.036 |
| PHQ-9 | 0.00 (0.00;0.00) | 0.00 (0.00;3.00) | Z = -2.111, P = 0.035 |
| PSQI | 2.00 (1.00;3.00) | 4.00 (3.00;6.00) | Z = -3.705, P ＜ 0.000 |
| HARQ | 6.93 (4.82) | 9.40 (6.00) | t=-1.754, P=0.085 |
| Capsaicin cough threshold |  |  |  |
| C2 (μmol/L) | 1.08(0.18) | 1.05 (0.16) | t=0.685, *P*=0.496 |
| C5 (μmol/L) | 1.17 (0.20) | 1.12 (0.19) | t=1.070, *P*=0.289 |
